# How do self‐efficacy and self‐concept impact mathematical achievement? The case of mathematical modelling

**DOI:** 10.1111/bjep.12443

**Published:** 2021-07-06

**Authors:** Mathias Holenstein, Georg Bruckmaier, Alexander Grob

**Affiliations:** ^1^ Department of Psychology Division of Developmental and Personality Psychology University of Basel Basel Switzerland; ^2^ University of Applied Sciences and Arts Northwestern Switzerland University of Education Institute of Secondary Education, Mathematics Education and Its Disciplines Windisch Switzerland

**Keywords:** self‐efficacy, self‐concept, mathematical modelling, mathematical achievement, school grades

## Abstract

**Background:**

According to the self‐enhancement perspective, self‐efficacy and self‐concept are shaped by prior achievement and have a crucial impact on future development. Their role in improving performance on challenging tasks, such as mathematical modelling (i.e., solving realistic problems mathematically), has barely been studied.

**Aims:**

We investigated patterns of self‐efficacy and self‐concept and their predictive effects on mathematical modelling while taking into account school grades as measure of prior achievement and reasoning to reveal cognitive and motivational effects on achievement.

**Sample:**

*N* = 279 secondary students in Grade 8 or 9 from 16 classes and 6 schools participated in the study.

**Method:**

The multi‐informant design consisted of teachers’ reports of school grades, students’ reports of self‐efficacy and self‐concept (questionnaire‐based), and assessment of students’ reasoning and mathematical modelling.

**Results:**

Using random‐intercept models, we found that the predictive effect of self‐efficacy on mathematical modelling withstood taking the school‐classroom‐related nested structure into account, whereas self‐concept lost its predictive value. Further, self‐efficacy fully mediated the effect of school grades on mathematical modelling.

**Conclusions:**

In line with the self‐enhancement perspective on self‐efficacy, our findings highlight the strength of motivational effects on mathematical modelling. When we take the nested structure into account, our results indicate an impact of school grades via self‐efficacy on mathematical modelling independent of students’ cognitive level or classroom. Given the diverse challenges such complex tasks present, important pedagogical and didactical recommendations, such as targeting the enhancement of students’ self‐efficacy by teachers and educational decision makers, can be drawn.

## Introduction

*Self‐efficacy –* believing in one’s capability to create an impact on current and future events and possessing the means to attain given goals (Bandura, 1977, 1997) – is crucial for students to realize their capabilities. Hence, self‐efficacy has been investigated as an important predictor of achievement (Bandura, 1997; Chemers, Hu, & Garcia, 2001; Kriegbaum, Jansen, & Spinath, 2015; Zimmerman, Bandura, & Martinez‐Pons, 1992). Building on these findings, researchers have studied the enhancement of self‐efficacy to improve students’ performance in general (Bandura, 1997; Multon, Brown, & Lent, 1991; Zimmerman et al., 1992) and mathematical achievement in particular (Prabawanto, 2018; Schunk, 1983; Schunk & Cox, 1986).

The relationship between self‐efficacy and achievement appears especially relevant for mathematics, a cognitively and emotionally challenging subject for students (Hackett & Betz, 1989). Self‐efficacy has been found to be an important motivational predictor of performance in mathematical problem solving (Pajares & Miller, 1995). When predicting school achievement, cognitive predictors – with intelligence considered the strongest among them – have also been investigated (Gottfredson, 2002; Roth et al., 2015). These studies indicate that to investigate the relationship between self‐efficacy and mathematical problem solving, intelligence needs to be taken into account.

In the academic motivation literature, *self‐concept* – often and historically defined as a person’s global perception of himself or herself (Shavelson, Hubner, & Stanton, 1976) but more recently discussed as being subject specific when referring to academic self‐concept (e.g., Marsh, 1990) – and self‐efficacy appear to be highly related constructs, and researchers oftentimes struggle to differentiate them (cf., Bong & Skaalvik, 2003). We differentiate between prospective and evaluative self‐efficacy – one’s belief about how *capable* one is of doing something – and retrospective and descriptive self‐concept – one’s belief about how *good* one is at doing something (Marsh et al., 2019; Pajares & Schunk, 2001) while attributing subject‐specific characteristics to both constructs.

There have been a few studies concerning the relationship to mathematical achievement of *both self‐belief variables*, self‐efficacy and self‐concept. For example, Marsh et al., (2019) found distinctive frame‐of‐reference effects. Frame‐of‐reference effects mean that self‐concept and self‐efficacy arise from comparing one’s evaluation or description in a specific subject with that of other students in the same subject (social comparison) as well as from comparing one’s evaluation or description in that subject with one’s own in other subjects (dimensional comparison). Such subject‐specific effects were different for self‐concept and self‐efficacy. In the same study, Marsh et al., (2019) rejected globality as a distinctive characteristic of the relation of self‐concept to self‐efficacy. Therefore, empirically disentangling the effects of self‐concept and self‐efficacy seems crucial when investigating effects on mathematical achievement because in doing so, subject‐specific relations become clearer (Burns, Crisp, & Burns, 2020).

Studies revealed that self‐efficacy contributed to later achievement when prior performance was taken into account (Bandura, 1997; Gore, 2006; Lee & Seo, 2021). Similar links to mathematics achievement were also found for self‐concept (e.g., Van der Beek, Van der Ven, Kroesbergen, & Leseman, 2017). In summary, previous research indicates that prior achievement and students’ self‐beliefs are strongly connected. Although research remains rather inconclusive on the role of self‐efficacy and self‐concept in learning mathematics, current literature highlights the relevance of mathematical modelling for students’ application of mathematics in everyday life (for an overview, see Greefrath & Vorhölter, 2016). We, therefore, investigated the motivational effects of self‐efficacy and self‐concept and their role in the relationship between prior achievement (i.e., school grades) and mathematical modelling while controlling for influences of intelligence.

## Theoretical background

### Mathematical modelling

When students face a task in mathematics where they cannot rely on previous mastery experiences, which Bandura (1997) described as the most influential source of self‐efficacy and which could be the case in mathematical modelling (Blum & Borromeo Ferri, 2009), they have to draw on external feedback. Mathematical modelling is described as ‘the process of choosing and using appropriate mathematics and statistics to analyze empirical situations, to understand them better, and to improve decisions’ (Common Core State Standards Initiative [CCSSI], 2010, p. 72). Related to the conceptual category of problem solving, mathematical modelling is classified as a primary and secondary school standard for mathematical practice and as one of eight principles of mathematical practice (CCSSI, 2010; also see Blum & Niss, 1991). In learning mathematical modelling, students often face a degree of complexity they are not used to in their regular training, which is why Blum and Borromeo Ferri (2009) emphasized that students need to be taught mathematical modelling specifically. The idea of students transferring previously acquired mathematical skills and relying on mastery experience is supported by the effect found for previous school grades and self‐efficacy on later achievement (Caprara et al., 2008).

**Figure 1:**
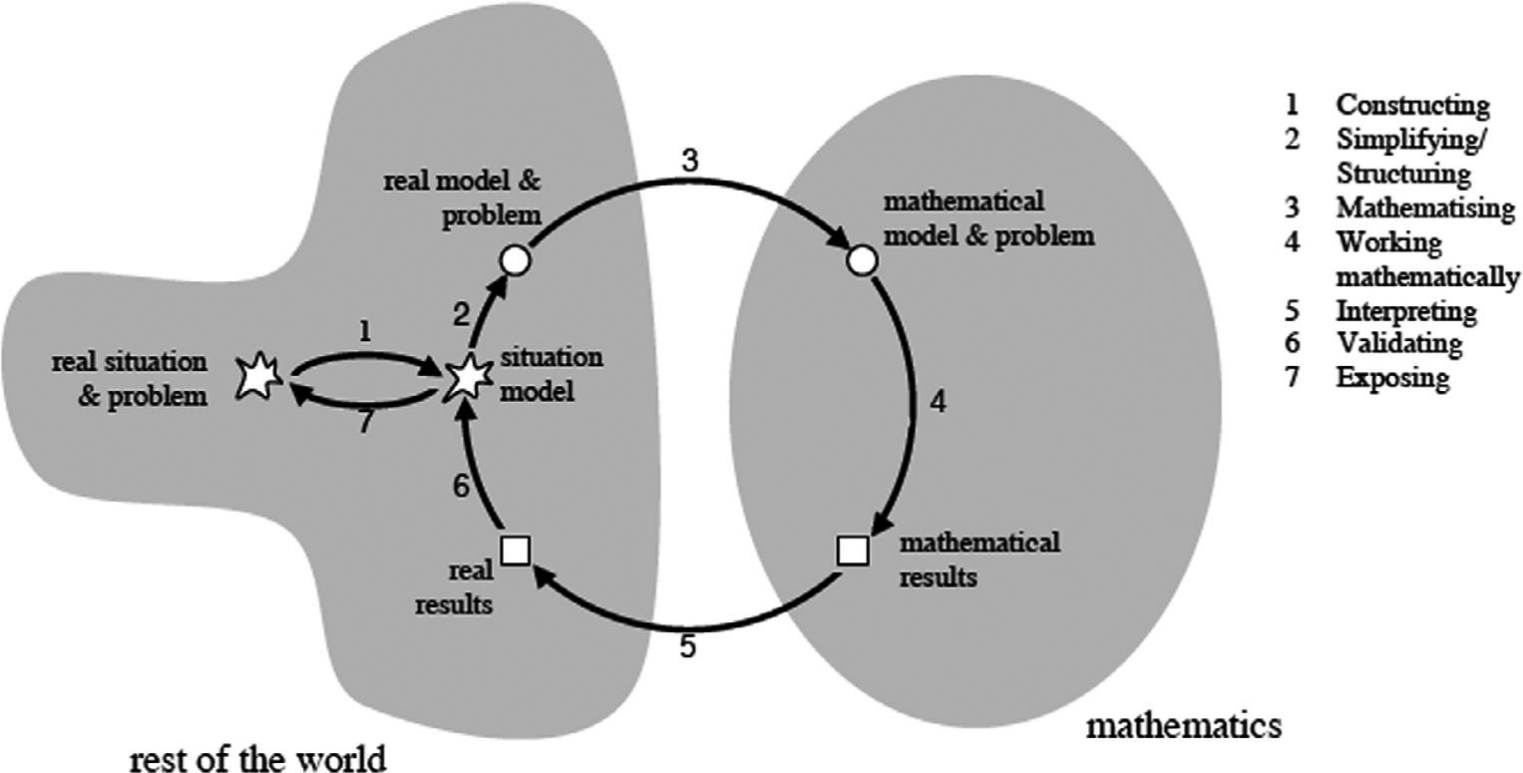
Modelling cycle (according to Blum & Leiss, 2007).

Research on teaching and learning mathematical modelling often draws on established conceptualizations of the modelling process as a multi‐step cycle. Figure 1 – as an example of one possible illustration (Blum & Borromeo Ferri, 2009; Blum & Leiss, 2007) – demonstrates that mathematical modelling is by definition linked to different mathematical and non‐mathematical competencies such as calculating, applying problem‐solving strategies, and reading and communicating (cf., Niss, 2003).

According to Leiss and Tropper (2014), solving realistic problems with mathematical means, that is, mathematical modelling, constitutes a crucial topic in the didactics of mathematics and is highly relevant for students’ learning of life skills. Surprisingly, few studies empirically support the assumption that skills acquired in mathematical modelling tasks transfer to real‐life problem solving, stressing the need for research in this domain (Brown & Stillman, 2017; Lehner et al., 2017). Theoretically, strong outcome effects on general problem solving would be assumed because (1) students make gains in underlying skill sets (cf., Baumert, Nagy, & Lehmann, 2012) and (2) mathematical modelling involves skills that differ from those needed for routine problems (Heinze, 2007).

### Predictors of mathematical modelling

Even more than geometry or calculus, mathematical modelling is considered especially difficult for students (Blum & Borromeo Ferri, 2009). These difficulties can vary depending on the phase of the modelling process (e.g., Stillman, Brown, & Galbraith, 2010). When learning mathematical modelling, students are encouraged to use metacognitive strategies such as reflecting on their activities to approach certain difficulties (Stillman, 2011), supporting the assumption that self‐efficacy plays a crucial role. Current literature reveals several *predictors* aside from self‐beliefs in general and self‐efficacy in particular. In addition to predictors that account for differences in achievement such as intelligence (Gottfredson, 2002; Roth et al., 2015), reasoning skills (Baumert, Brunner, Lüdtke, & Trautwein, 2007), and reading comprehension (Borromeo Ferri, 2006; Leiss, Schukajlow, Blum, Messner, & Pekrun, 2010; Phonapichat, Wongwanich, & Sujiva, 2014), some predictors seem to be specifically relevant to mathematical modelling. Prior mathematical skills such as counting (Aunola, Leskinen, Lerkkanen, & Nurmi, 2004) and calculation (Andersson, 2007) as well as results of mathematics tests (Leiss et al., 2010; Marsh et al., 2018) and self‐beliefs (Pajares & Miller, 1994; Schukajlow, Achmetli, & Rakoczy, 2019) have likewise been found to be linked to mathematical modelling.

Predictors of mathematical modelling become relevant at different steps in the modelling cycle (cf., Figure 1). For instance, prior mathematical skills such as counting and calculation influence how students solve a mathematical problem in the mathematical world. In this regard, self‐beliefs might influence mathematical modelling on different levels depending on the step at which they get activated: for example, when students build the situation model, when they calculate the numbers, or even at the very beginning when they approach the task. In this way, self‐beliefs contribute uniquely and substantially to mathematical modelling (cf., Schukajlow et al., 2019). The combination of its important position in educational standards and the fact that many students struggle with mathematical modelling stresses the need to look more deeply at *predictors of mathematical modelling* in efforts to help students improve their mathematical modelling. Looking at the effects of self‐beliefs, namely, self‐efficacy and self‐concept, can provide such insights.

### Relationship of self‐efficacy and self‐concept to mathematical modelling

Social‐cognitive learning theory, which takes a self‐enhancement perspective (Bandura, 1977), postulates that self‐efficacy has a positive effect on students' ability to make use of their competencies and, therefore, predicts their achievement. Using path analysis, Pajares and Miller (1994) found a predictive effect of mathematical self‐efficacy on mathematical problem solving. Together with a study by Pajares and Graham (1999), medium to strong positive coefficients resulted for the relationship between self‐efficacy and mathematical achievement. Recent research found that students’ beliefs in their capability to solve mathematical modelling tasks were low compared to other mathematical challenges such as ‘dressed‐up’ word problems and intra‐mathematical problems (Krawitz & Schukajlow, 2018).

Czocher, Melhuish, and Kandasamy (2019) showed in an interventional study that mathematical self‐efficacy could be improved with a mathematical modelling competition. Moreover, an investigation of interventions with specific modelling techniques targeting enhancement of self‐efficacy in relation to perceived competence found that especially students who created multiple solutions gained from higher self‐efficacy (Schukajlow et al., 2019). Taken together, empirical evidence highlights that mathematical self‐efficacy is a crucial predictor of mathematical modelling.

We believe it is necessary to empirically disentangle the effects of self‐concept and self‐efficacy when investigating how self‐beliefs influence mathematical modelling. Meißner, Greiff, Frischkorn, and Steinmayr (2016) revealed that students' self‐concept affected mathematical problem solving differently depending on the measurement when comparing achievement tests with school grades. Marsh et al., (2018) found that mathematical self‐concept was predictive of test scores and school grades. Moreover, self‐concept measures seemed to have higher predictive value than self‐efficacy measures for later school grades and test scores in mathematics (Marsh et al., 2019). In these studies that conceptually differentiate between retrospective and descriptive aspects (i.e., self‐concept) of self‐beliefs on the one hand and prospective and evaluative ones (i.e., self‐efficacy) on the other, the authors found discriminable effects when both constructs were operationalized using subject‐specific items. In contrast, Bong and Clark (1999) found that evidence for their predictive utility was more consistent for self‐efficacy than self‐concept though the direction remains unclear. More recently, consistent support was found for a reciprocal relationship to skill development for both self‐concept and self‐efficacy (Burns et al., 2020), indicating a dependence regarding the operationalization of self‐belief constructs.

### Relationship of school grades to mathematical modelling

Although many studies have explored the difficulties in fostering students' mathematical modelling when teaching mathematics, little is known about the influence of prior achievement. For the prediction of general mathematical competency, a study using data from the Program for International Student Assessment (PISA; Organisation for Economic Co‐operation & Development [OECD], 2019) revealed prior competence as the most important longitudinal predictor of mathematical achievement (Kriegbaum et al., 2015). The authors further found that intelligence was the best cross‐sectional predictor and self‐efficacy was the strongest motivational predictor. Considering the strong effect social comparison has on students’ achievement (cf., the big‐fish‐little‐pond effect, Marsh & Seaton, 2015), it can be assumed that prior achievement, in many studies operationalized as school grades, also plays a crucial role in how students achieve in mathematical modelling tasks. Not surprisingly, prior mathematical skills account for much of students' mathematical modelling competency or, stated conversely, the lack of such skills for students' difficulties (Andersson, 2007; Aunola et al., 2004; Leiss et al., 2010). Given these findings, one might argue that part of the connection between prior achievement and mathematical modelling can be explained by the effect that school grades, as a strong source of achievement feedback, have on students' self‐concept and self‐efficacy.

Meißner et al. (2016) found different predictive effects of self‐concept measures on problem‐solving abilities as an outcome when compared to school grades as an outcome although the two outcome variables were intercorrelated. Whereas for mathematical problem solving, cognitive abilities combined with self‐concept were of predictive value, for school grades the impact of self‐concept on its own was stronger. Examining a large longitudinal study over 6 years at the start of secondary school, Marsh et al., (2018) found reciprocal predictive effects of mathematical self‐concept, mathematical test scores, and school grades. Reciprocal effects between school grades and self‐concept measures, which in these studies partly overlap our definition of self‐efficacy, were consistently found for school achievement in general (e.g., Marsh & O’Mara, 2008) and mathematical achievement in particular (Julie, 2020; Niepel, Brunner, & Preckel, 2014). In most studies, school grades were treated as outcome variables for mathematical problem solving (e.g., Wüstenberg, Greiff, Vainikainen, & Murphy, 2016) but so far they have not been investigated as predictors. However, regarding the assumed reciprocal effects of prior achievement, self‐concept, self‐efficacy, and mathematical modelling, school grades should also be considered *predictors* of mathematical modelling when arguing that they constitute an important source of students’ self‐beliefs.

### Self‐efficacy mediating the relationship of school grades to mathematical modelling

Taking Bandura's (1977) self‐enhancement perspective, self‐beliefs can be perceived as *mediators* in the relationship between school grades and mathematical modelling. Research on achievement development in general showed that especially self‐efficacy acts as a mediator between achievement and predictors such as positive emotions (Oriol‐Granado, Mendoza‐Lira, Covarrubias‐Apablaza, & Molina‐López, 2017), classroom environment (Tosto, Asbury, Mazzocco, Petrill, & Kovas, 2016), test accommodations (Einav, Sharabi, Tal Even‐hen, & Margalit, 2018), cognitive activation strategies (Li, Liu, Zhang, & Liu, 2021), or, as described above, mathematical test scores and school grades (Marsh et al., 2019). While various studies have hinted that self‐efficacy serves as an important mediator in the effects of predictors of school achievement and at the same time is treated as a crucial covariate of mathematical modelling, little is known about how self‐efficacy acts as mediator in the relationship between school grades, mathematical achievement, and especially mathematical modelling. Further, how the effects of the two self‐belief variables on mathematical modelling differ remains unclear.

## Aims of the present study and hypotheses

Research on issues related to mathematical modelling, such as mathematical problem solving, indicates that prior achievement, in many studies operationalized as school grades, is of predictive value to mathematical modelling (Julie, 2020; Marsh et al., 2018; Niepel et al., 2014). However, because self‐concept and self‐efficacy are widely perceived as being influenced by the feedback students receive on their performance (Burns et al., 2020; Marsh et al., 2019; Meißner et al., 2016) and because school grades constitute an important feedback source for students (Bandura, 1977), we presumed that self‐concept and self‐efficacy play a crucial role in the assumed predictive effect of school grades on mathematical modelling (see Hypothesis 1a and b). We therefore investigated how school grades, mathematical self‐concept, and mathematical self‐efficacy predict mathematical modelling.

While research so far hinted that mathematical self‐concept tends to be a better predictor of outcomes such as later school grades and test scores than self‐efficacy (Marsh et al., 2019), we assumed that the reverse is true for a more immediate achievement measure, which is the case for mathematical modelling (cf., Bong & Clark, 1999). This result would be in line with Kriegbaum et al., (2015), who found self‐efficacy to be the strongest motivational predictor of mathematical competence on the PISA (OECD, 2019). Following this argument, we presumed that students especially need to believe in their capability in a prospective, evaluative sense, that is, believe in their self‐efficacy (rather than the retrospective, descriptive evaluation, i.e., relying on their self‐concept), when confronted with modelling tasks that are especially challenging for them and structurally new to them. This idea is supported by Kriegbaum et al., (2015), who argued that self‐efficacy items (compared to self‐concept items) are more closely aligned with achievement tasks and explained their finding with the level of specificity of self‐efficacy. Moreover, and in addition to the prospective, evaluative sense of self‐efficacy, the task‐specificity of self‐efficacy implies a criterion‐based comparison rather than a social one (Bong & Skaalvik, 2003), which, again, accounts for the assumption of a stronger connection of mathematical modelling with self‐efficacy than with self‐concept. Taken together, these studies inspired us to investigate how self‐concept and self‐efficacy predict mathematical modelling (see Hypothesis 1a and b). We assumed self‐efficacy would be more strongly connected to our outcome for theoretical and measurement‐related reasons (see Hypothesis 2).

Concerning school grades, we assumed that prior achievement in terms of feedback given by teachers, namely, grades when investigated on its own would do little to explain students' performance in mathematical modelling tasks when controlling for reasoning skills and accounting for effects of the reference group, namely, the respective class. Instead, considering the mediating role of self‐efficacy in the relationship of achievement to different outcome variables (e.g., Li et al., 2021; Marsh et al., 2019), we assumed that self‐efficacy would act as a mediator in the effect of school grades on mathematical modelling (see Hypothesis 3). This, again, we assumed would be the case only for mathematical self‐efficacy because of its specificity, alignment, and prospective orientation, which mathematical self‐concept lacks.

We postulated the following hypotheses:

Hypothesis 1a Mathematical self‐efficacy predicts mathematical modelling.

Hypothesis 1b Mathematical self‐concept predicts mathematical modelling.

Hypothesis 2 When investigated together, mathematical self‐efficacy will more strongly predict mathematical modelling than mathematical self‐concept.

Hypothesis 3 Mathematical self‐efficacy mediates the effect of school grades on mathematical modelling.

To better investigate our assumption on the role of mathematical self‐efficacy in contrast to mathematical self‐concept, we also looked at potential mediating effects of the latter. Given the strong connection of intelligence and achievement (Gottfredson, 2002; Roth et al., 2015), we targeted predictive and mediating effects above influences of students’ cognitive level and, therefore, included students’ reasoning skills as a control variable in the analyses. Because school grades play a decisive role in determining students’ future, they become highly important in the transition from secondary school to higher education or work life (e.g., Ogg, Zimdars, & Heath, 2009). It is at this stage of students’ life when the predictive value of school grades should be examined thoroughly. We, therefore, investigated our hypotheses in the secondary school years. Besides looking at students’ grade in mathematics, which is assumed to be the primary source of achievement feedback for the mediating role of school grades on mathematical modelling, we included grade in German as a control variable in this analysis because language skills have also been shown to be predictive for mathematical modelling (Leiss et al., 2010; Phonapichat et al., 2014).

## Method

### Sample

For our analyses, 279 students (53.9% girls, missing information for gender = 8) from 16 classes in six schools in Switzerland were assessed as part of a larger research project in spring 2020. Written consent that highlighted the voluntariness of participation was obtained from all participants. Students had a mean age of 15.1 years (*SD* = 0.68; ranging from 13.3 to 16.8 years, missing information for age = 7) and were attending their eighth (*n* = 123) or ninth (*n* = 156) school year (see United Nations Educational, Scientific, & Cultural Organization Institute for Statistics, 2013). Most federal states in Switzerland differentiate between two secondary school levels: one follows a basic vocational orientation (basic requirements) while the other has an advanced vocational and academic orientation (advanced requirements). Sixty‐seven students (24.0%) were allocated to basic requirements and 212 students (76.0%) followed advanced requirements, which is fairly representative of the distribution in Switzerland.

### Measures

To answer our research questions, information was collected from different sources: direct test assessment of students' mathematical modelling and reasoning skills, self‐assessment of students’ self‐efficacy and self‐concept in a questionnaire, as well as students’ school grades from their official school certificate reported by their teachers. Teachers were instructed to conduct test assessments, to administer the questionnaire, and to send back all material to be analysed by the project. Participation altogether took two school lessons’ time in the students’ regular timetable.

#### Mathematical modelling

Two tests with five mathematical modelling tasks each were put together, resulting in parallel versions with items being similar regarding mathematical content and solution path. Modelling tasks were adopted from a pool originally established for the DISUM^1^ project (e.g., Schukajlow, Kolter, & Blum, 2015), for example, ‘Mr. Flower wants to get to Zurich airport by taxi. In the daily newspaper he finds offers from two cab companies: *Gerard's Taxi Fleet*, 2.10 Swiss francs base fee, 1.60 Swiss francs per kilometre; *Taxi Taxi*, no base fee, 1.75 Swiss francs per kilometre. With which cab company should he get to the airport? Explain your answer carefully’.

A student's answer on each of the five tasks was rated as correct or incorrect and a sum score (conceptually ranging from 0 to 5) was calculated. In effect, the maximum in our sample was 4 because no student managed to solve all five tasks correctly. Together with a mean of *M* = 1.62 (*SD* = 1.16), this reflects the difficulty of unfamiliar mathematical modelling tasks (cf., Blum & Borromeo Ferri, 2009). Cronbach’s alphas for the two test versions were .34 and .48, respectively. Rather, low Cronbach’s alphas might be due to the construct's conceptually broad range (cf., modelling cycle), low number of items, heterogeneity of contexts and answer format, or the complexity and nonroutine aspects of such tasks. Relying on good reliabilities found in previous studies using these tasks (Leiss et al., 2010; Schukajlow et al., 2009), sufficient reliability is assumed for the two tests in our study.

#### Reasoning

Four subtests of the Testing System for Scholastic and Educational Counselling, Grades 6 to 13 – Revised (German language version: PSB‐R 6‐13; Horn, Lukesch, Mayrhofer, & Kormann, 2003) were administered to assess reasoning. The PSB‐R 6‐13 is a standardized intelligence assessment consisting of numerical, visuo‐spatial, and verbal subtests and was designed for educational settings. In the present study, we used the reasoning subscale containing four subtests: numerical, literal, and figural series as well as conception of spaces, yielding a Cronbach’s alpha of .66.

#### Mathematical self‐efficacy

A scale consisting of four items from the PISA (OECD, 2019) was used to assess mathematical self‐efficacy, which followed our definition in an evaluative and prospective sense of how *capable* one is of something (see the Introduction). A mean score was calculated from answers on a 5‐point Likert scale; a sample item is ‘In mathematics, I am certain of being able to understand the most difficult topics.’ Cronbach's alpha for this scale was .90.

#### Mathematical self‐concept

To assess students' mathematical self‐concept, an adaption of the Academic Self‐Description Questionnaire (ASDQ; Marsh, 1990) was used, which followed our definition in a descriptive and retrospective sense of how *good* one is at something (see the Introduction). Students were given a 4‐point Likert scale to answer six items on their self‐concept in mathematics (e.g., ‘I am good at mathematics’), for which a mean score was calculated. Cronbach’s alpha was .93.

#### School grades

Teachers reported students' half‐year grades in mathematics and language (German) from their official school certificate as a measure of their prior achievement. Grades in Switzerland range from 1 to 6, in most cases effectively ranging from 3 (*insufficient*) to 6 (*very good*), and are given in half‐grade intervals.

### Data analysis

All analyses were conducted using R (R Development Core Team, 2008). To treat missing values that were mainly due to students being absent on the respective measurement occasion, we imputed these values using the Multivariate Imputation by Chained Equations package (mice; van Buuren & Groothuis‐Oudshoorn, 2011). For preliminary analyses on the hypotheses, bivariate correlations were calculated. To account for the nested structure of the data, especially regarding effects of the reference group, that is, the classroom, in relation to school grades, we then ran random‐intercept models using the lme4 package (Bates, Mächler, Bolker, & Walker, 2015); that is, the respective intercepts could vary between classrooms. To compute *p* values for random‐intercept models, we used the Tests in Linear Mixed Effects Models package (lmerTest; Kuznetsova, Brockhoff, & Christensen, 2017). We ran mediation analyses on these models with the mediation package (Tingley, Yamamoto, Hirose, Keele, & Imai, 2014).

## Results

### Descriptive statistics

Descriptive statistics for all variables analysed and correlations with gender as well as among all variables are shown in Table 1. No significant correlations were found with students’ age. Correlations with gender were found for grade in German (girls had better grades), mathematical self‐efficacy, and mathematical self‐concept (both lower for girls). Moreover, significant correlations were found among most variables of interest, especially between mathematical self‐efficacy and self‐concept (*r* = .75, *p* < .001). With regard to this relationship, incremental effects are accounted for by simultaneously including both variables in the same analysis (see next section). Reasoning correlated positively with all other variables; the same was true for the math grade. Moreover, grade in German correlated with mathematical modelling (*r* = .17, *p* < .01), which was expected because of the need for language and reading comprehension skills. No significant correlations were found for grade in German with self‐concept or self‐efficacy, presumably because these were assessed in a subject‐specific way. We, therefore, included gender, reasoning, and grade in German as control variables for the following analyses.

**Table 1:** Minima, maxima, means, standard deviations, and correlations with gender for reasoning, grades in German and mathematics, mathematical self‐efficacy, mathematical self‐concept, and mathematical modelling; manifest correlations among all variables

Variable	*n*	Min	Max	*M*	*SD*	Gender	Reasoning	German grade	Mathematics grade	Mathematical self‐efficacy	Mathematical self‐concept
Reasoning	255	21.00	79.00	49.41	8.92	.10					
German grade	274	3.00	6.00	4.73	0.49	.34***	.24***				
Mathematics grade	274	3.00	6.00	4.66	0.60	−.02	.33***	.36***			
Mathematical self‐efficacy	260	1.00	5.00	3.16	0.97	−.20***	.24***	.04	.45***		
Mathematical self‐concept	261	1.00	4.00	2.68	0.84	−.22***	.27***	.05	.64***	.75***	
Mathematical modelling	260	0.00	4.00	1.62	1.16	.00	.27***	.17**	.24***	.24***	.24***

Note

*N* = 279. Descriptive statistics with complete data on the respective scales; correlations with gender as well as among variables of interest with imputed data. Gender coding: 1 = male, 2 = female.

* *p* < .05, **p < .01, ***p < .001.

### Multilevel analyses

In Table 2, a random‐intercept model is displayed that was calculated to take the nested structure of the data, that is, classroom and school level, into account. When classroom levels were controlled and variables of interest examined simultaneously, a significant predictive effect was found for mathematical self‐efficacy but not for mathematical self‐concept on mathematical modelling. This finding confirmed only Hypothesis 1a and not Hypothesis 1b, meaning that only self‐efficacy predicted mathematical modelling while self‐concept did not. Further, Hypothesis 2 was confirmed: mathematical self‐efficacy more strongly predicted mathematical modelling than mathematical self‐concept. The relationship of reasoning with mathematical modelling lost its significance when the nested structure was considered. We did not include grades in this model because these relations were examined in the following analyses on Hypothesis 3.

**Table 2:** Fixed effects of mathematical self‐efficacy and mathematical self‐concept predicting mathematical modelling, controlling for gender and reasoning

Variable	Standardized coefficients Beta (β)
(Intercept)	.03
Mathematical self‐efficacy	.17*
Mathematical self‐concept	.07
Gender	−.01
Reasoning	.08

Note

*N* = 279.

* *p* < .05.

**Figure 2:**
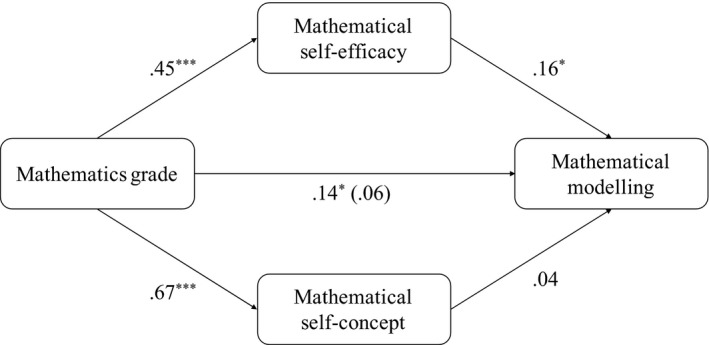
Mediation analyses for mathematical self‐efficacy and mathematical self‐concept mediating the effect of mathematics grade on mathematical modelling with fixed effects (*z*‐standardized), controlling for gender, reasoning, and grade in German.

To test for Hypothesis 3, we ran random‐intercept models, again to account for values nested in classrooms, to investigate mediating effects of mathematical self‐efficacy and self‐concept on the relationship between mathematics grade and mathematical modelling. The corresponding mediation model is displayed in Figure 2. We included gender, reasoning, and grade in German as control variables in these analyses.

We first tested for the predictive effect of mathematics grade on mathematical modelling (*r* = .14, *p* < .05), which appeared to be significant. We then tested the predictive effects of mathematics grade on mathematical self‐efficacy (*r* = .45, *p* < .001) and on mathematical self‐concept (*r* = .67, *p* < .001). Finally, we ran a model with mathematics grade predicting mathematical modelling and both mediators, that is, mathematical self‐efficacy and self‐concept, which revealed a significant predictive effect of mathematical self‐efficacy on mathematical modelling (*r* = .16, *p* < .05). No significant predictive effect was found for mathematical self‐concept (*r* = .04) while the effect of mathematics grade on mathematical modelling lost its significance (*r* = .06). In summary, a mediation effect of mathematical self‐efficacy could be assumed.

Using bootstrapping procedures with 1,000 samples, we tested the significance of the indirect effect of mathematics grade through mathematical self‐efficacy on mathematical modelling. The bootstrapped indirect effect was.07 (*p* < .05), confirming Hypothesis 3: The effect of mathematics grade on mathematical modelling was fully mediated via mathematical self‐efficacy. With the corresponding analysis, no mediation effect was found for mathematical self‐concept.

## Discussion

The aim of this study was to investigate mathematical self‐efficacy and mathematical self‐concept as motivational predictors of mathematical modelling and, moreover, as mediators for effects of school grades on mathematical modelling. In an extension of previous research on self‐beliefs and mathematical modelling, we assumed that mathematical self‐efficacy plays a crucial role in effects that prior achievement measured by school grades are shown to have on mathematical modelling. We found that students' mathematics grade influenced both self‐efficacy and self‐concept to a high degree, indicating that self‐beliefs are shaped through the feedback learners receive. Further, we intended to shed light on the distinction between mathematical self‐concept and self‐efficacy, both constituting students' self‐beliefs at the secondary school level.

While significant bivariate correlations hinted at predictive effects of self‐efficacy and self‐concept on mathematical modelling, our results revealed that only self‐efficacy was predictive of mathematical modelling when taking the nested structure of the data into account. Following social learning theory (Bandura, 1997), we claim that this finding is due to a potential self‐enhancement effect of self‐efficacy on a more immediate measure such as mathematical modelling. Students need to believe, prospectively, in their potential to achieve something (i.e., their self‐efficacy) rather than relying on whether they, retrospectively, think they are good at something (i.e., their self‐concept). This is in line with recent findings on motivational predictors of achievement showing that self‐efficacy acts as a strong predictor aside from the influence of cognitive skills such as intelligence (Kriegbaum et al., 2015).

Our results revealed self‐efficacy as a predictor of mathematical modelling and as a mediator in the relationship between mathematics grade and mathematical modelling irrespective of students' school grades in German. Including language as well as mathematical skills is especially important considering that, in view of the modelling cycle, students' language skills were previously found to be linked to mathematical modelling (e.g., Holenstein et al., 2020) through reading comprehension (Leiss et al., 2010; Vilenius‐Tuohimaa, Aunola, & Nurmi, 2008) or understanding context (Borromeo Ferri, 2006; Phonapichat et al., 2014). Regarding reasoning, the influence on mathematical modelling was diminished when taking the nested structure into account. By looking only at descriptive statistics, a positive relationship can be assumed, which would be in line with theoretical arguments claiming that reasoning and modelling share underlying skill sets (e.g., Baumert et al., 2007). We argue that some variance in reasoning was lost when students were assigned to different school levels. Further, we found that the relationship of mathematics grade, a form of previous achievement feedback, and mathematical modelling also seemed to be dependent on the respective classroom. When the nested structure was taken into account, a connection between grades and mathematical modelling became less clear, whereas mathematical self‐efficacy served as a mediator and shed light on this relation.

Finally, full mediation was found for mathematical self‐efficacy for the effect of school grades on mathematical modelling. This is in line with arguments in previous studies that self‐efficacy independently contributes to academic achievement and is more than a simple reflection of prior performance (Bandura, 1997; Caprara et al., 2008; Pajares & Schunk, 2001). We argue that students rely on prior achievement feedback given by teachers in different domains – in our study mathematics and German – in building their self‐efficacy, which affects their mathematical modelling performance. Keeping in mind that at the same time grades were found to be highly class dependent (cf., Fang et al., 2018), serious implications can be concluded.

### Practical implications

Our results reveal a predictive effect of mathematical self‐efficacy on mathematical modelling, extending previous research on predictors of mathematical development. In this regard and following the self‐enhancement perspective (Bandura, 1977), improving self‐efficacy constitutes an opportunity to help students foster their mathematical modelling independent of their skill level. We advise teachers, firstly, to scrutinize the self‐beliefs of their protégés in order to be especially aware of students with low self‐efficacy and then, secondly, to integrate supportive, benevolent elements into their teaching to help enhance students’ motivation when new tasks such as mathematical modelling are introduced; our findings suggest that students' performance might profit in return.

Keeping in mind the lack of causal evidence our results contain, a reverse impact might occur when students show progress in self‐efficacy by receiving positive feedback on mathematical modelling. We therefore, thirdly, advise teachers to make sure that mathematical modelling tasks are taught in a way that allows for individual levels and that students understand how these tasks can be solved. Taking different steps of the modelling cycle into consideration (Blum & Borromeo Ferri, 2009) and supporting learning by applying a solution plan (Schukajlow et al., 2015) are promising approaches in this regard.

Considering the mediating role of self‐efficacy in the effect of school grades on mathematical modelling, we examined grades as a feedback source. In line with previous research (Fang et al., 2018; Wößmann & West, 2006), we found in our study that grades were highly dependent on the respective classroom. With regard to the big‐fish‐little‐pond effect (Marsh & Seaton, 2015) and the effects grades are supposed to have on mathematical self‐efficacy (Marsh et al., 2018), one could argue that grades have a negative effect on some students' self‐beliefs. Assuming that there is some causality in the effects of school grades on later achievement mediated by self‐efficacy, students receiving low grades might get into a downward spiral that is more likely a consequence of low grades rather than of their level in mathematical development. We therefore, fourthly and again, encourage teachers to provide feedback in a way that positively affects students’ self‐beliefs. In sum, basing feedback on individual progress rather than social comparison might be in order.

### Limitations of the current study and directions for future research

Although our study design includes some longitudinality by assessing students’ grades from the previous semester and later assessing students’ self‐assessment and testing their mathematical modelling, our results mainly rely on cross‐sectional data. Therefore, the validity of causal effects is theoretically assumed, which is supported by previous studies hinting at reciprocal relationships (e.g., Burns et al., 2020), which would imply bidirectional causality between self‐beliefs and mathematical achievement. Concerning intellectual preconditions, note that we relied solely on reasoning, whereas verbal dimensions might also contribute to mathematical modelling especially because of the requirements of reading comprehension. Predictive effects of school grades and mathematical self‐efficacy are strengthened by controlling for reasoning and self‐concept measures. Nevertheless, future studies should aim to follow longitudinal designs that take a look at long‐term (potential co‐)development of self‐efficacy, self‐concept, and mathematical achievement. Moreover, interventional studies are needed to challenge the practical implications of improving students’ self‐efficacy beliefs in order to turn the downward spiral into a virtuous circle.

Looking at our measures, some limitations lie in the mathematical modelling tasks and the corresponding rating we used. While we used 10 items established for the DISUM project (e.g., Schukajlow et al., 2015), more mathematical modelling tasks are needed to investigate broader aspects of different phases of mathematical modelling to shed light on specific challenges students struggle with on such tasks. Regarding scales on self‐beliefs, we used established measures from PISA (OECD, 2019) and the ASDQ (Marsh, 1990) focusing on subject‐specific assessment. We encourage future researchers to investigate discrete forms of self‐beliefs in different domains or more general conceptualizations when investigating such complex tasks as mathematical modelling, where various skills and challenges play a decisive role. Considering today’s variety of possibilities for evaluating students’ academic achievement, following a multi‐informant design as we did with test assessment, questionnaire information, and teacher reports holds promising chances to broaden the understanding of the effects of school grades, self‐beliefs, and academic achievement.

## Conflicts of interest

All authors declare no conflict of interest.

## Author contribution

Alexander Grob (Conceptualization; Supervision; Writing – review & editing) Georg Bruckmaier (Conceptualization; Supervision; Writing – original draft; Writing – review & editing) Mathias Holenstein (Conceptualization; Formal analysis; Funding acquisition; Investigation; Methodology; Project administration; Writing – original draft; Writing – review & editing).

## Data Availability

The data that support the findings of this study will be openly available on The Open Science Framework (OSF) upon acceptance for publication.

## Footnotes

^1^
Didactical Intervention Modes for Mathematics Teaching Oriented Towards Self‐Regulation and Directed by Tasks.
